# A Case Report of Female Hydrocele of the Canal of Nuck (Type I): A Diagnostic Challenge and Surgical Solution

**DOI:** 10.7759/cureus.47926

**Published:** 2023-10-29

**Authors:** Fotios Gkioulos, Sofia Theodoridou, Berk Abay, Alec H Engledow

**Affiliations:** 1 General Surgery, Barts Health NHS Trust, London, GBR; 2 Critical Care, Barts Health NHS Trust, London, GBR

**Keywords:** case report, inguinolabial swelling, groin swelling, hernia, hydrocele of canal of nuck

## Abstract

The canal of Nuck is an anomaly of the female inguinal canal that can lead to the development of hernias or hydroceles. It is a result of incomplete obliteration of a pouch of the parietal peritoneum that accompanies the round ligament throughout the inguinal canal. This is a case report of a 27-year-old female with hydrocele of the canal of Nuck which was initially misdiagnosed as a reducible right inguinal hernia. Physical examination and ultrasound revealed a right groin 64 × 15 × 36 mm cystic mass extending from the right inguinal region to the labia majora. Right inguinal exploration was performed with an oblique inguinal incision. A cystic lesion measuring 65 × 15 mm was carefully dissected from the round ligament and excised. Histopathological examination confirmed the diagnosis of hydrocele of the canal of Nuck. The patient is doing well after six months with no signs of recurrence on the operated side. The hydrocele of the canal of Nuck, though a rare condition, should always be considered in the differential diagnosis when evaluating inguinolabial swellings in female patients.

## Introduction

The canal of Nuck is an anomaly of the female inguinal canal that can lead to the development of hernias or hydroceles. Normally, the inguinal canal contains the spermatic cord in males and the round ligament of the uterus in females. During early embryonic development, the pouch of the parietal peritoneum accompanies the round ligament throughout the inguinal canal, typically undergoing obliteration within the first year of life. This structure is homologous to that of the processus vaginalis in males [1]. Failure to obliterate this evagination of the parietal peritoneum can result in an indirect inguinal hernia or hydrocele of the canal of Nuck. Given its rarity (0.76% rate in a large series of 787 inguinal surgical explorations) [2] and limited clinical awareness, misdiagnosis (e.g. inguinal hernia, femoral hernia, lipoma, inguinal lymph node, tumors, etc.) and inappropriate treatment are common occurrences. In this report, we present an interesting case involving a 27-year-old female with hydrocele of the canal of Nuck, initially misdiagnosed as a reducible right inguinal hernia. Subsequently, the patient underwent elective surgical excision of the hydrocele.

## Case presentation

**Figure 1:**
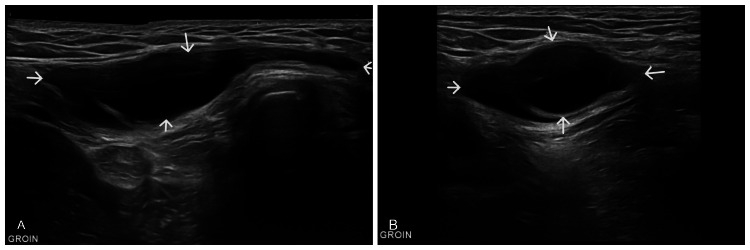
Ultrasound scan showing an anechoic cystic tubular structure (arrows) in the right groin measuring 64x15x36mm.

A 27-year-old female was referred to our outpatient general surgery clinic by a general practitioner (GP) as a case of a reducible right inguinal hernia. Eight months prior, the patient noticed a small swelling in her right groin area, initially attributed to a possible infection. At that time, the patient did not experience any significant symptoms. Importantly, swelling was not observed during infancy or adolescence. However, the patient’s condition deteriorated over the course of six months. The pain became severe, preventing her from attending work. She sought medical attention in the Accident & Emergency (A&E) department because of increasing pain in the affected area and progressive enlargement of the swelling, which varied with her body position (increasing while standing and reducing while in a supine position). After evaluation by multiple A&E physicians during her attendance, she was discharged with a reducible right inguinal hernia diagnosis. The patient reported no history of trauma to the area, no changes in bowel or bladder habits, and no significant medical or surgical history. Upon examination at our clinic, we palpated a 4 × 2 cm cystic lump in the right groin area which was fluctuant, non-tender, non-adherent to the skin, with no obvious skin changes and it was partially reducible. We considered the possibility of a hydrocele in the canal of Nuck. Ultrasonography (US) of the right groin confirmed a 64 × 15 × 36 mm cystic mass extending from the right inguinal region to the labia majora (Figure 1).

**Figure 2:**
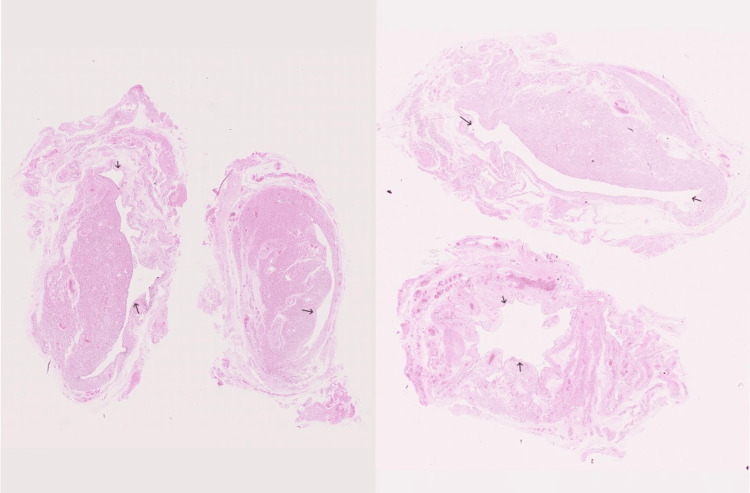
Histopathology - Hydrocele of the Canal of Nuck.

Subsequently, the patient underwent elective excision of the Nuck canal. We made an oblique incision in the right groin during the procedure, revealing a cystic lesion measuring 65 × 15 mm. This lesion extended from the peritoneal cavity through the inguinal canal to the right labia majora. We carefully dissected the lesion from the round ligament and ligated the canal near the deep inguinal ring. No inguinal hernia was observed. The surgical wound was closed in layers, and the patient experienced an uneventful postoperative recovery with no evidence of recurrence in the last six months. Histopathological examination revealed a cystic space lined by mesothelium surrounded by connective tissue, consistent with the excision of the canal of Nuck - Type I (Figure 2).

## Discussion

The canal of Nuck, first described by Dutch anatomist Anton Nuck in 1691, plays a pivotal role in the development of the inguinal canal in both male and female fetuses. This canal is formed by two structures: the gubernaculum and processus vaginalis (begin to form approximately in the eighth to 12th week of gestation) [3]. The gubernaculum is a ligament attached to the developing gonad that extends to the fetal groin. Shortening and thickening of the gubernaculum facilitate the descent of gonads in males, whereas in females, the gubernaculum attaches to the uterine cornua. The caudal portion becomes the round ligament connecting the uterine cornua to the labia majora, contributing to the normal positioning of the uterus and preventing ovarian descent into the inguinal canal. The processus vaginalis emerges during the first trimester as a peritoneal evagination, herniating through the inguinal canal of the abdominal wall [4]. In females, the portion of the processus vaginalis within the inguinal canal is termed the canal of Nuck, which typically obliterates in a craniocaudal direction between the eighth month of gestation and one year of life. The patent canal of Nuck is normal during the first year but poses an anatomical risk for hernia or hydrocele development. The canal of Nuck usually consists of two layers: an outer layer of varying fibrous thickness, and an inner wall composed of single-layered mesothelial cells. Hydrocele formation occurs when there is an imbalance between fluid secretion and absorption through the secretory membrane of the inner wall. This imbalance may result from inflammation, infection, reduced lymphatic drainage, or injury [5]. The hydrocele of the canal of Nuck is classified into three distinct types, each characterized by specific anatomical features and clinical presentations [6].

Type I - encysted type (most common)

Type I, often referred to as the "encysted" type, represents the most prevalent form of hydrocele in the canal of Nuck. In this type, there is a lack of communication between the cyst and peritoneal cavity due to obliteration of the proximal part of the canal. The cyst can be localized at any point along the course of the round ligament, extending from the deep inguinal ring to the labia majora. Typically, cysts of this type are relatively small, measuring less than 3 cm in size. However, their size can vary and may suddenly increase owing to factors such as infection, trauma (leading to intracystic bleeding), or impaired lymphatic drainage. Clinically, Type I hydroceles may present as irreducible tender lumps in the groin, occasionally leading to misdiagnosis as an incarcerated hernia [6].

Type II

In Type II hydroceles, there is no obliteration in the proximal part of the canal, resulting in persistent communication between the peritoneal cavity and hydrocele sac. This communication can lead to variations in the size of the hydrocele depending on the patient's position. The clinical diagnosis of Type II hydroceles may involve specific maneuvers, such as the Valsalva maneuver or changes in the patient's standing position, to aid in the assessment [6].

Type III (uncommon)

Type III represents the least common form of hydrocele in the canal of Nuck. In this variant, constriction occurs at the deep inguinal ring, creating a formation resembling an hourglass. In Type III, the proximal part of the canal is intraperitoneal, while the distal part remains within the inguinal canal. This unique anatomical configuration distinguishes Type III from other types and may present distinct clinical challenges during examination and diagnosis. 

Understanding these classification types is crucial for the accurate diagnosis and appropriate management of hydroceles of the canal of Nuck, as each type may necessitate different clinical approaches and considerations. Clinical examination of the hydrocele of the canal of Nuck poses challenges due to the limited literature and clinician knowledge. The findings typically include a painless or moderately tender lump in the inguinolabial region. Differential diagnoses encompass a wide range of conditions, including inguinal hernia, femoral hernia, lipoma, inguinal lymph node, vascular aneurysms, tumors (neurofibroma of the ilioinguinal nerve, leiomyomas, etc.), endometriosis of the round ligament, epidermal cysts, and childhood asymmetric labium majus enlargement (CALME) [6].

Establishing a diagnosis of hydrocele of the canal of Nuck based solely on history and clinical examination is challenging. As we discussed in our case making the diagnosis based only on clinical examination findings was very challenging and prone to making the wrong diagnosis. Thus, using different imaging investigations, particularly ultrasound, is invaluable for differentiation and diagnosis. On ultrasound, a patent canal of Nuck is a normal finding during the first year of life and serves as an anatomical risk factor for hernias. An encysted Type I hydrocele typically appears as an anechoic collection with posterior enhancement and no color Doppler flow surrounded by a thin wall. Hypoechoic or low-level echoes may be present owing to protein content. A "cyst-in-cyst" appearance may occur, resembling a small, thin-walled cyst within the primary cyst, occasionally resembling a follicular ovary [6]. Surgical excision is recommended when cysts become symptomatic, as aspiration often results in temporary relief and fluid reaccumulation.

Surgical management of Type II communicating hydrocele of the canal of Nuck typically involves high ligation of the processes vaginalis. In some cases, particularly in pediatric patients, it may be advisable to consider waiting until the child reaches the age of two years before performing surgical repair because there is a relatively high chance of spontaneous obliteration of the hydrocele during early childhood [7].

## Conclusions

The hydrocele of the canal of Nuck, though a rare condition, should always be considered in the differential diagnosis when evaluating inguinolabial swellings. Relying solely on history and clinical examination for diagnosis poses significant challenges owing to the rarity of the condition and varied clinical presentations. In our case the initial diagnosis given by the A&E physicians was inguinal hernia, which can be easily mistaken clinically, and requires different surgical approach. Hence, a high index of suspicion is crucial when examining the affected areas and knowledge of the differential diagnosis when examining inguinolabial swellings. The judicious use of imaging modalities such as ultrasound plays a pivotal role in confirming the diagnosis and guiding appropriate management. In cases where hydrocele of the canal of Nuck is diagnosed and symptomatic, surgical excision is the preferred treatment approach. Timely surgical intervention can effectively address this condition, mitigate morbidity, and prevent potential complications. A well-informed and comprehensive approach to diagnosis and management ensures the best possible outcomes for patients with this uncommon, yet clinically significant condition.
